# Evaluation of lipidomic change in goat sperm after cryopreservation

**DOI:** 10.3389/fvets.2022.1004683

**Published:** 2022-10-20

**Authors:** Bingbing Xu, Ruijun Wang, Zhiying Wang, Hongfu Liu, Zhen Wang, Weihang Zhang, Yanjun Zhang, Rui Su, Zhihong Liu, Yongbin Liu, Jinquan Li, Jiaxin Zhang

**Affiliations:** ^1^College of Animal Science, Inner Mongolia Agricultural University, Hohhot, China; ^2^Key Laboratory of Animal Genetics, Breeding and Reproduction, Hohhot, China; ^3^Key Laboratory of Mutton Sheep Genetics and Breeding, Ministry of Agriculture and Rural Affairs, Hohhot, China; ^4^Research Center for Animal Genetic Resources of Mongolian Plateau, College of Life Sciences, Inner Mongolia University, Hohhot, China; ^5^Inner Mongolia Jinlai Animal Husbandry Technology Co., Ltd., Hohhot, China

**Keywords:** lipidomic, buck spermatozoa, cryotolerance, phosphatidylcholine, triglyceride

## Abstract

The current study aimed to detect the relationship between the spermatozoa cryotolerance and the post-thawed sperm lipidome. Ejaculates from 20 goats, and performed a uniform frozen-thawed procedure in this study. According to the total motility of thawed sperm of goats, semen samples were classified into HF group (High Freezers, *n* = 8) with >60% total motility and LF group (Low Freezers, *n* = 8) with < 45% total motility. The lipidomic analysis based on UHPLC-MS/MS was utilized to investigate the relationship between sperm cryotolerance and their lipid metabolites expression. The results showed that the cryotolerance of sperm from different individual goats were in great variation. The total motility of post-thawed sperm in HF group (60.93 ± 2.43%) is significantly higher than that in LF group (34.04 ± 3.41%, *P* < 0.01). And the post-thawed sperm in HF group exhibited significantly higher plasma membrane (59.06 ± 2.34%) and acrosome integrity (62.93 ± 1.15%) than that in LF group (34.06 ± 4.85%, 44.92 ± 2.19% respectively, *P* < 0.01). The total of 29 lipid subclasses and 1,133 lipid molecules in the post-thawed goat sperm were identified by lipidomics analysis. The lipid content of thawed sperm in HF group was higher than that in LF group, the lipid profile in HF group was significantly separated from LF group, which indicated that the difference in lipid composition and lipid metabolism mode of sperm between the two groups was existed, especially the expression of phosphatidylcholine and triglyceride molecules. In conclusion, the cryotolerance of sperm from different individual goats were in great variation. Sperm with high cryotolerance may be able to uptake more lipids during cryopreservation. The increase in phosphatidylcholine and triglyceride content of thawed. Sperm may relate to more active lipid anabolic processes.

## Introduction

Artificial insemination can utilize the male animals with fine genetic properties effectively, and rapidly increase the proportion of excellent individuals in population (1, 2). Semen cryopreservation can prolong the life of sperm, and is frequently used in artificial insemination (1–4). However, sperm undergo cold shock, ice crystal formation, oxidative stress, and other molecular changes during cryopreservation, resulting in significant freezing damage on sperm motility, viability, and structural integrity (5–7). Compared with fresh semen, the fertilization capacity of sperm after cryopreservation was reduced. Therefore, it will limit the applications of frozen-thawed semen for artificial insemination in goats (6, 8–10).

However, variations have been observed in the quality of semen from different individuals after frozen-thawed procedures, these were influenced by the lipid composition of the sperm plasma membrane, the freezing protocol, and species-specific characteristics (11–16). The plasma membrane is an extremely important structure for sperm physiology as it regulates the critical events of sperm cholesterol efflux and calcium influx during oocyte-sperm interactions (17–19). Injury to the plasma membrane can trigger cell death and loss of homeostasis (20, 21). Thus, the biochemical composition of the plasma membrane is an important topic in sperm physiology and cryopreservation. The sperm plasma membrane is assumed to be the main structure in which sperm freezing damage occurs (22). Such damage results in membrane instability caused by lateral lipid rearrangement and membrane lipid loss (23, 24), and has potential impacts on sperm motility, osmotic stress response, and capacitation (25–28). Goat sperm are more sensitive to the cryopreservation process than those of other domestic species, possibly owing to lipid changes in the plasma membrane of goat sperm during cryopreservation (29). In addition, some studies have reported that sperm cells take up lipids or fatty acids from their environment during *in vitro* incubation (30–32).

Generally, there has been a lack of consistent cryopreservation protocols for different individual. The sperm motility of goats after thawed is still not good. Thus, it is necessary to further address the limitations of the goat semen cryopreservation technique. In this study, we performed uniform freeze-thawed process to goat sperm from different individuals, and the lipidome variation among semen samples with different quality after thawed were revealed by lipidomic analysis. All of these will provide a research basis and possible future directions for improving the goat semen cryopreservation technology.

## Materials and methods

### Semen collection, cryopreservation and thawing

Semen samples were provided by the Inner Mongolia Jinlai Livestock Technology Co., Ltd. (TuZuo country, Hohhot, Inner Mongolia, China). Twenty male Inner Mongolian cashmere goats at ages of 2 years old were selected, and the semen in each individual was collected three times a week during the reproductive season. The semen volumes in each sample ranged from 0.75 to 1.5 mL. The semen concentration was measured by A Bovine Accuread Photometer (IMV, France). The criteria for semen samples used for subsequent analysis are as follows: concentration of 20–30 × 10^8^ sperm/mL, and percentage of sperm total motility ≥75%.

Except for antibiotics, all chemicals used in this study were purchased from Sigma-Aldrich (St. Louis, MO, USA). The collected fresh semen was diluted with preheated a Tris-citric-glucose base solution at the volume ratio of 1:5 at 37°C, and centrifugally washed at 600 g at 4°C for 3 min, this process was repeated twice to remove the seminal plasma. Then, the washed sperm were extended with freezing diluent at 37°C, semen concentration was adjusted to 2 × 10^8^ sperm/mL, equilibrated at 4°C for 3 h. The freezing diluent were composed of 300 mM Tris, 95 mM citric acid, 56 mM glucose, 10% (v/v) egg yolk, 5% (v/v) glycerol, and 1% (v/v) antibiotics (Gibco). The chilled semen was loaded into 0.25 mL straws, and exposed to liquid nitrogen vapor at a height of 4 cm for 7 min. The straws were then placed into liquid nitrogen for storage. After 2 days of storage, straws were thawed in a water bath at 37°C for 30 s. The thawed semen was centrifuged at 10,000 g for 3 min, and the pellet was washed twice before being subjected lipidomic analysis.

### CASA-motility evaluation

The sperm motility was assessed soon after thawing. A 3 μL thawed semen was dropped into a prewarmed Leja slide analysis chamber. A computer-assisted semen analyzed system (CASA, IVOS II, IMV, France) was applied to measure sperm motion parameters at 250 × magnification. A minimum of five fields were evaluated per sample, and a minimum of 1,000 sperm were counted. The main sperm motion parameters measurements included total motility (TM; %), progressive motility (PM; %), curvilinear velocity (VCL; μm/s), straight line velocity (VSL; μ/s), average path velocity (VAP; μ/s), linearity (LIN, %) and the amplitude of lateral head displacement (ALH; μ). The definitions of these sperm motility parameters can be found in Dorado (33), and the customized settings used for goat sperm are shown in Table 1.

**Table 1:** Setting for goat semen analyses.

**Features**	**Setting**
Frame capture (Hz)	60
Frame count	30
Head size max (μm^2^)	70
Head size min (μm^2^)	6
Elongation max (%)	100
Elongation min (%)	1
Slow VAP (μm/s)	20
Slow VSL (μm/s)	30
Max photometer	70
Min photometer	60
Progressive STR	80
Progressive VAP (μm/s)	30
Static VAP (μm/s)	4
Static VSL (μm/s)	1
Head brightness min	200
Tail brigthness min	96
Maximum width to length ratio (%)	90
Minimum width to length ratio (%)	1
Temperature (°C)	37

### Flow cytometric analysis

The fluorescence signals of marked sperm were analyzed by using a flow cytometer (ACEA NovoCyteTM, Agilent, USA) equipped with a 488 nm blue all-solid-state laser, a 640 nm red all-solid-state laser, and a photomultiplier tube. The applied fluorescence channels (FITC, PE, PerCP, and APC) were monitored simultaneously. The recorded parameters included the area of all channels, width, height, and time. These parameters were used to adherent cells, cell debris and single cells to be effectively distinguished. A total of 20,000 events were analyzed for each semen sample.

The plasma membrane integrity status of the frozen-thawed sperm was evaluated using Annexin V (AV) and propidium iodide (PI, BD Biosciences, USA) following the protocol described by Peña (34). Each semen aliquot was diluted in 1 × binding buffer, supplied with the kit, to a concentration of 1.2 × 10^6^ cells/mL. Fluorescent dyes (5 μL AV and 5 μL PI; 50 μg/mL) were added to the sperm suspension to enable identification of cell events, following incubation in the dark, and sperm were washed in PBS. The AV/PI results revealed four sperm subpopulations: live sperm (AV–/PI–), live sperm with damaged plasma membrane (AV+/PI–), dead cells (AV+/ PI+), and dead late-necrotic cells (AV–/PI+).

The acrosome status of the frozen-thawed sperm was assessed using FITC-peanut (Arachis hypogaea) agglutinin (GENMED SCIENTIFICS, INC. USA). Briefly, frozen-thawed semen was diluted to 1.2 × 10^7^ cells/mL, and mixed with 150 μL FITC-PNA and 200 μL PI (0.4 μg/mL). The mixed suspensions were incubated in a darkened area for 15 min (35). After staining, the FITC-PNA signal (green) was read as an intact acrosome marker, and dead sperm were indicated by the PI signal (red). Readings were performed on each sample, and four sperm populations were identified using FITC/ PerCP filters: sperm particles, dead sperm, intact sperm and sperm with damaged acrosome.

### Sample preparation and lipid extraction

The methyl tert-butyl ether (MTBE) technique was used to extract lipids. In brief, samples were homogenized using 240 μL methanol and 200 μL water. Following the addition of 800 L MTBE, the mixture was subjected to ultrasonication for 20 min at 4°C before being left undisturbed remaining still for 30 min at room temperature. The solution was centrifuged at 14,000 g for 15 min, at 10°C, and the upper organic solvent layer was collected and dried under nitrogen.

### LC-MS/MS method for lipid analysis

A CSH C18 column (1.7 μm, 2.1 mm × 100 mm, Waters) was used for LC separation utilizing reversed-phase chromatography. The lipid extracts were re-diluted in 200 μL of a 90/10 mixture of isopropanol and acetonitrile and centrifuged at 14,000 g for 15 min, after which 3 μL of the sample was injected later. Acetonitrile-water (6:4) and acetonitrile-isopropanol (1:9), both with formic acid and ammonium formate contents of 0.1% and 0.1 Mm, served as solvents A and B, respectively. At a flow rate of 300 L/min, the initial mobile phase of solvent B contained 30% of its volume. It was maintained for 2 min, increased linearly to 100% solvent B over 23 min, and then equilibrated in 5% solvent B for 10 min.

Q-Exactive Plus was used to acquire positive and negative mode mass spectra. All measurements used the following optimized preset ESI parameters: ion spray voltage 3,000 V; S-Lens RF level 50%; source temperature 300°C, and capillary temperature was 350°C. The scan range of the instruments was 200–1,800 m/z.

### Data processing and analysis

“Lipid Search” is a search engine for identifying lipid species based on MS/MS math. In the LipidSearch database, there are more than 1,500,000 fragment ions and more than 30 lipid classes. The precursor and fragment mass tolerances were both set to 5 ppm. Peak identification, peak extraction, and lipid identification were all performed using LipidSearch (secondary identification). The precursor tolerance is set at 5 ppm, the product tolerance is set at 5 ppm, and the product ion threshold is set at 5%.

Univariate statistical analysis, multivariate statistical analysis, and hierarchical cluster analysis were performed on the data retrieved by LipidSearch. Student's *t*-test, a non-parametric test, and multiplicity of variation analysis were included in the univariate statistical analysis. The multivariate statistical analysis included orthogonal partial least square discriminant analysis (OPLS-DA). The variable importance in projection (VIP) scores were evaluated, and the metabolites with VIP >1 were chosen as class discriminants.

### Statistical analysis

The difference significance of kinetics and flow cytometry variables of all samples between the HF and LF groups were analyzed using Student's *t*-test in SPSS statistical software (version 22.0; Chicago, IL, USA). And the data are presented as mean ± SEM.

## Results

### Identification of goats with HF or LF and sperm evaluation

**Figure 1:**
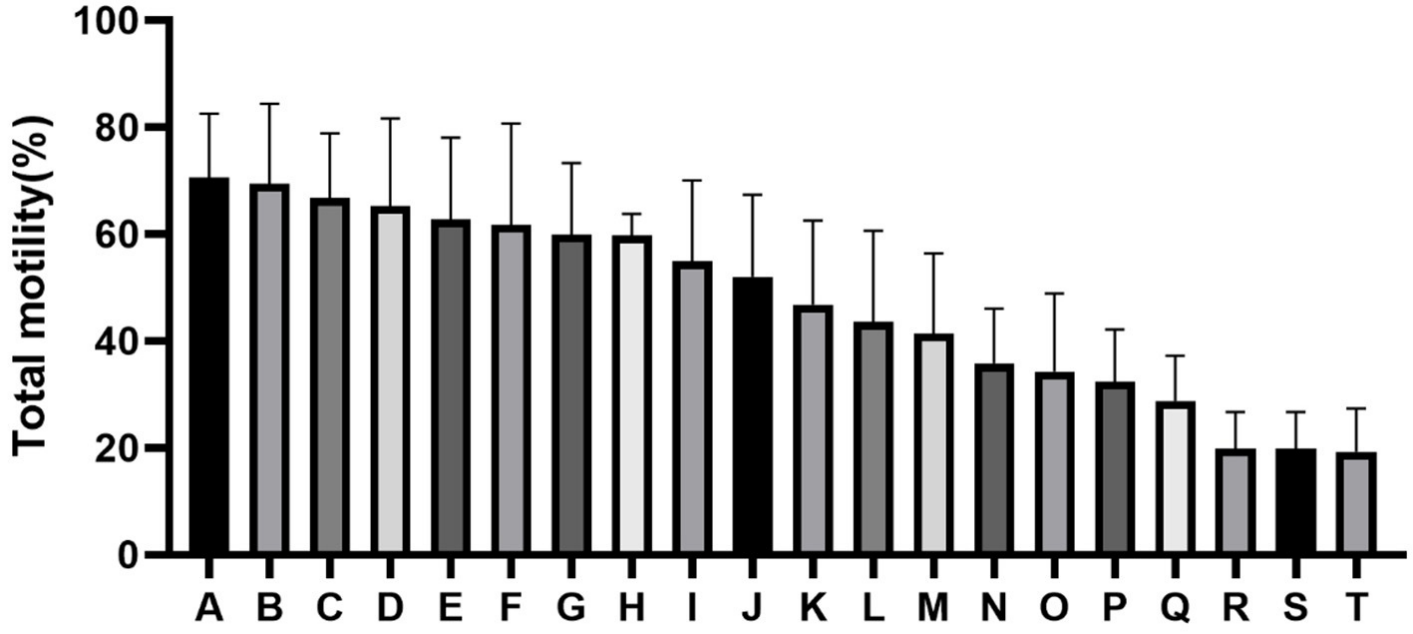
Difference in percentage total motility as determined by computer-aided sperm analysis (CASA) assessment of spermatozoa from all goats (A-T).

**Figure 2:**
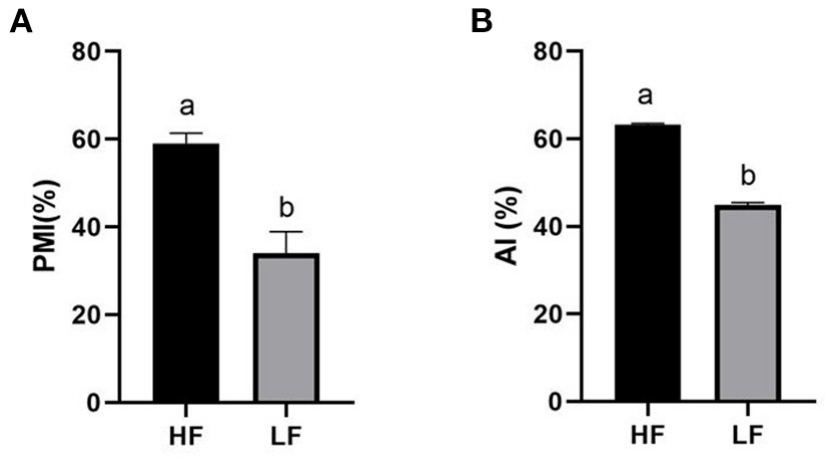
Differences of sperm parameters. Percent differences in the levels of **(A)** PMI, plasma membrane integrity, **(B)** AI, acrosome integrity. The data are presented as mean±SEM. (*P* < 0.05, calculated using Student's *t*-test).

The quality of frozen-thawed sperm varied among the individuals. The frozen-thawed sperm samples from different goat individuals were sorted based on total motility (Figure 1), the eight samples with the higher total motility (>60%) were as HF group, and the another eight samples with the lower motility (< 45%) were as LF group, and the motion characteristics showed significant differences between HF and LF (*P* < 0.01, Table 2), all of the motion parameters in HF group was significant higher than that in LF group. The results showed that sperm plasma membrane integrity and acrosome integrity were higher in the HF group than that in the LF group (*P* < 0.01, Figures 2A,B).

**Table 2:** Differences in the motion kinematics between HF and LF.

**Parameter**	**HF**	**LF**
TM (%)	60.93 ± 2.43^a^	34.04 ± 3.41^b^
PM (%)	31.91 ± 2.94^a^	17.46 ± 1.67^b^
VAP (μm/s)	45.22 ± 2.61^a^	23.76 ± 4.14^b^
VSL (μm/s)	36.98 ± 2.59^a^	19.36 ± 2.94^b^
VCL (μm/s)	74.49 ± 5.85^a^	41.22 ± 9.24^b^
LIN (%)	30.74 ± 2.51^a^	15.75 ± 3.24^b^
ALH (μm)	3.84 ± 0.33^a^	2.23 ± 0.51^b^

Data presented as mean±SEM. (A) TM, total motility; (B) PM, Progressive motility; (C) VAP, Average path Velocity; (D) VCL, Curvilinear Velocity; (E) VSL, Straightline Velocity; (F) ALH, Amplitude of Lateral Head Displacement; (G) LIN, Linearity. Different superscript lowercase letters indicate extremely significant differences, P < 0.05.

### Identification of lipid class and species quantity

**Figure 3:**
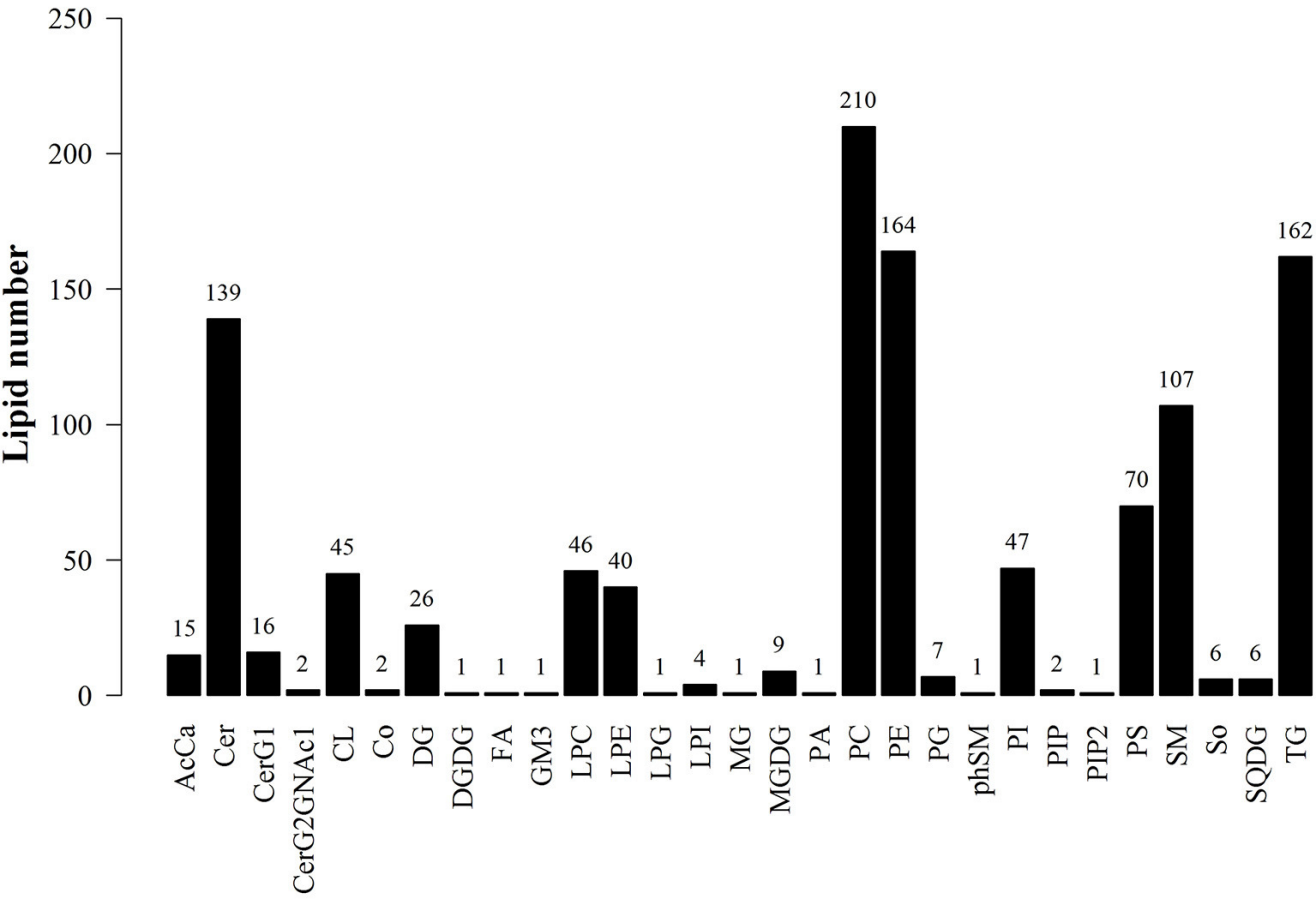
Statistics of lipid subclasses and lipid molecules. AcCa (Acyl Carnitine), Cer (ceramides), CerG1 (glucocerebroside), CerG2GNAc1 (Simple Glc series), CL (cardiolipins), Co (Coenzyme), DG (Triacylglycerol), DGDG (digalacyosyl diacylglycerols), EPA (Eicosapentaenoic acid), FA (fatty acid), GM3 (Gangliosides), LPC (lyso-Phosphatidylcholine), LPE (lyso-Phosphatidylethanolamine), LPG(lyso-phosphatidylglycerols), LPI (lyso-phosphatidylethanola), MG (monoacylglycerol), MGDG (monogalactosyldiacylglycerols), PA (phosphatidic acids), PE (phosphatidylethanola mines), PG (phosphatidylglycerols), PhSM (phytosphingosine), PI (phosphatidylethanola), PIP (phosphatidylinositol (4)phosphate), PIP2 (phosphatidylinositol (4,5)bisphosphate), PS (phosphatidylserines), SM (sphingomyelin), So (Sphingosine), SQDG (Sulfoquinovosyldiacylglycerol), TG (triglyceride).

**Figure 4:**
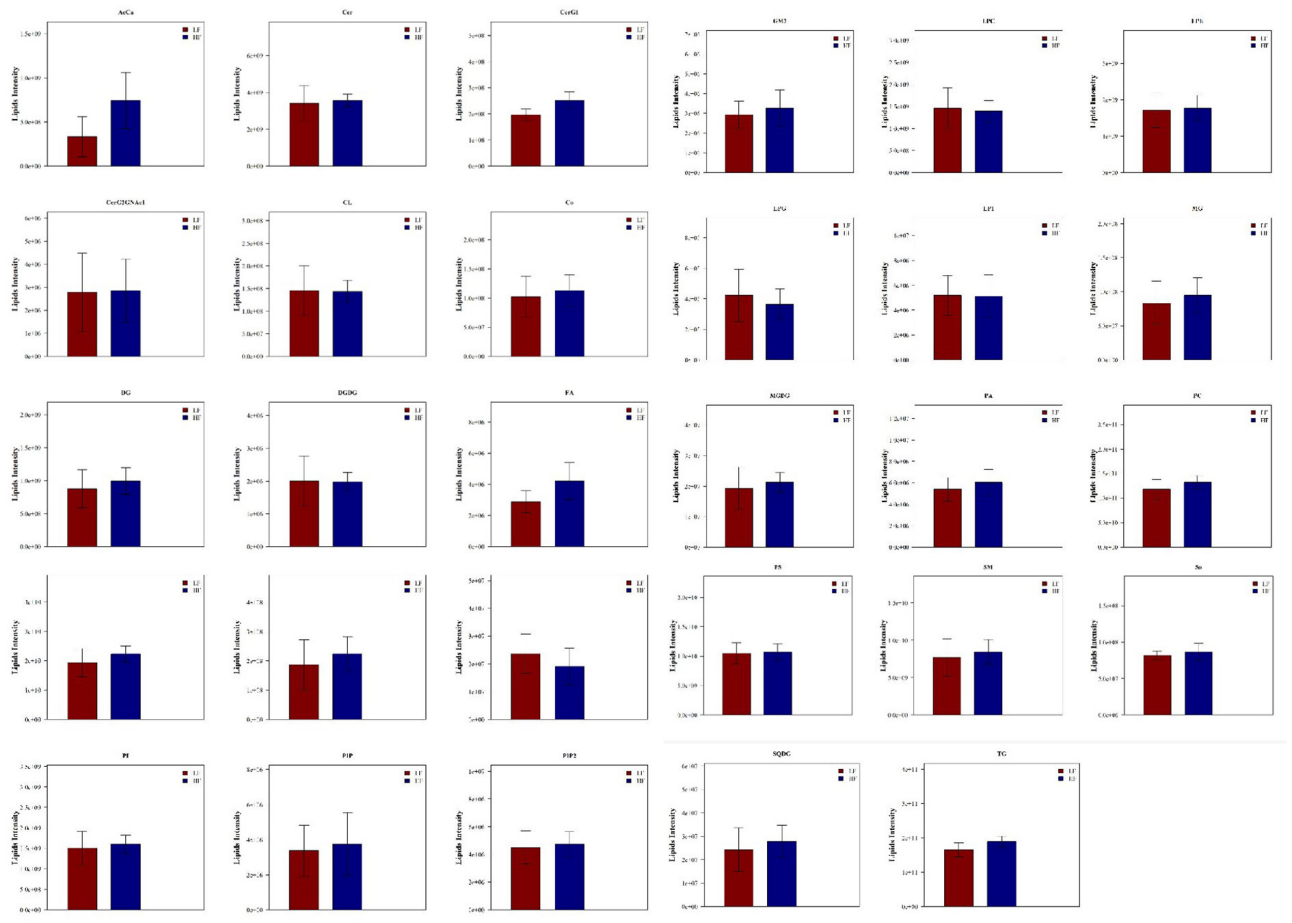
Relative abundance of lipid subclasses between HF and LF group.

LipidSearch was used to analyze the data in both positive and negative modes in this study. The lipid classes and the number of lipids identified in each class are shown in Figure 3. It was found that 29 lipid classes and the number of 1,133 lipids species were identified. Phosphatidylcholine, phosphatidylethanolamine, triglyceride, ceramides, sphingomyelin were observed as the major lipid classes in the goat sperm. The total lipid content in HF sperm was higher than that in LF sperm, and the relative abundance of each lipid subclass in HF and LF groups is shown in Figure 4.

### Multivariate statistical analysis

**Figure 5:**
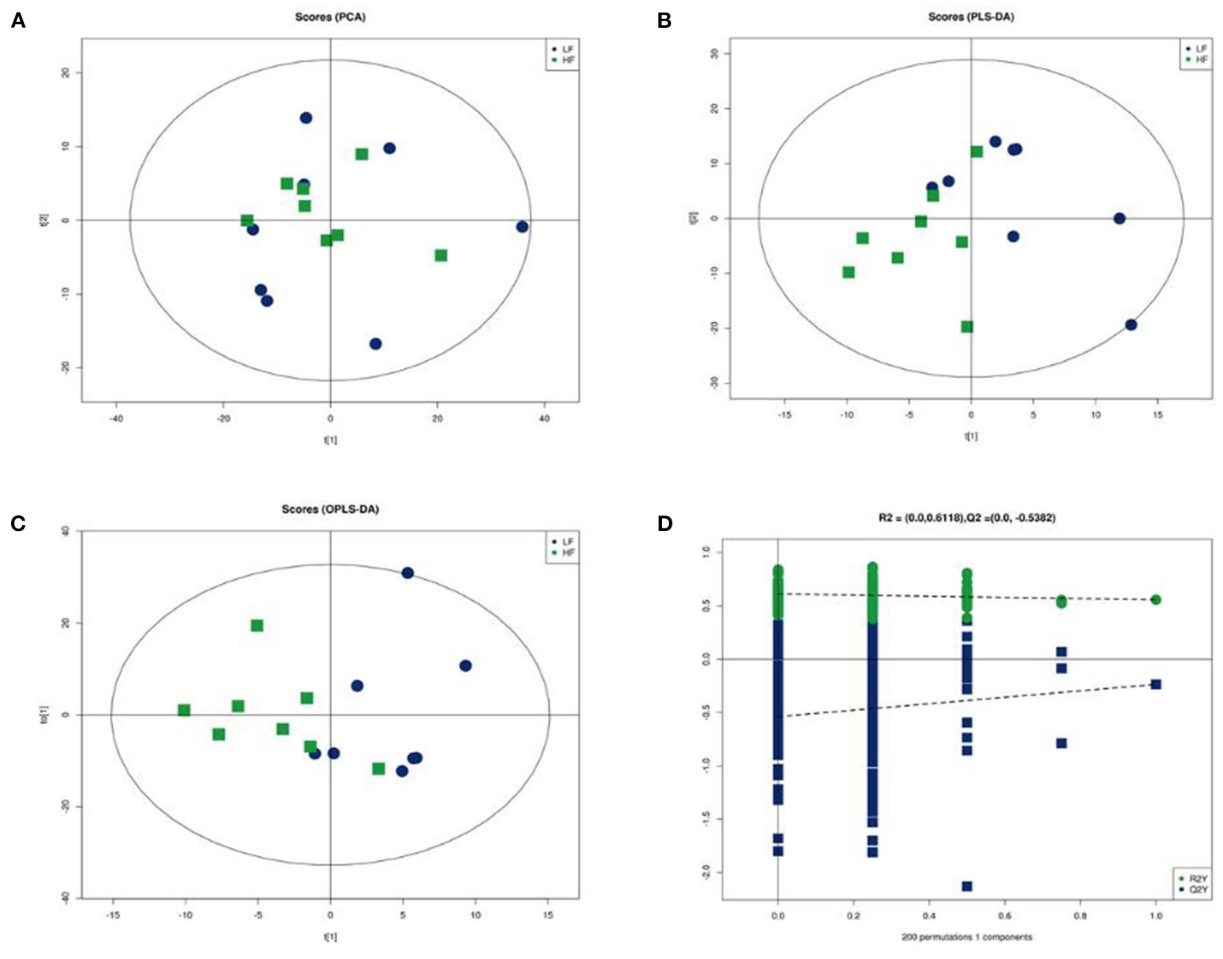
Multivariate statistical analysis of seminal plasma metabolic profiling between HF and LF. **(A)** Principal component analysis score plot. **(B)** Partial least squares-discriminant analysis score plot. **(C)** Orthogonal partial least squares-discriminant analysis (OPLS-DA) score plot. **(D)** Statistical validation of the established OPLS-DA model with permutation analysis (200 random permutations).

The clear segregation of lipidomic profiles between HF and LF was not observed using principal component analysis (PCA). Further supervised analysis, PLS-DA and OPLS-DA were conducted using the training set (Figure 5). The performance characteristics of the PLS-DA and OPLS-DA models were R2(X) = 0.575; R2(Y) = 0.557; and Q2(Y) = −0.723. The permutation test (200 random permutations) validated the model, and no overfitting of the data was observed. According to PLS-DA score plots, the HF and LF groups were separated into two distinct clusters.

The potential of the lipid species as a marker was assessed by the VIP score for the PLS-DA model. Variables with VIP scores >1 was regarded as significant for the classification model. The phosphatidylcholine and triglyceride lipid species showed significant difference between the HF and LF groups (Table 3), and they showed a higher abundance in the HF group.

**Table 3:** The differential lipid species in high and low freezability sperm.

**Lipid ion**	**Lipid group**	**Ion formula**	**m/z**	**RT- (min)**	**FC**	***P*-value**	**VIP**
PC (31:1) + H	PC (31:1) + H	C39 H77 O8 N1 P1	718.54	11.43	0.82	0.035	7.58
PC (16:0/16:1) + H	PC (32:1) + H	C40 H79 O8 N1 P1	732.55	10.18	0.71	0.012	6.99
PC (16:0/18:1) + H	PC (34:1) + H	C42 H83 O8 N1 P1	760.59	11.15	0.86	0.026	6.86
TG (16:0/14:0/16:1) + NH4	TG (46:1) + NH4	C49 H96 O6 N1	794.72	19.39	0.60	0.009	6.84
TG (16:0/14:1/18:2) + NH4	TG (48:3) + NH4	C51 H96 O6 N1	818.72	18.35	0.76	0.035	6.53
TG (16:0/14:0/18:2) + NH4	TG (48:2) + NH4	C51 H98 O6 N1	820.74	19.44	0.66	0.036	6.24
TG (16:0/14:0/18:1) + NH4	TG (48:1) + NH4	C51 H100 O6 N1	822.75	20.58	0.74	0.014	5.60
TG (16:0/16:0/16:0) + NH4	TG (48:0) + NH4	C51 H102 O6 N1	824.77	21.62	0.81	0.012	5.01
TG (16:0/16:1/18:2) + NH4	TG (50:3) + NH4	C53 H100 O6 N1	846.75	19.55	0.77	0.047	4.81
TG (16:0/16:1/18:1) + NH4	TG (50:2) + NH4	C53 H102 O6 N1	848.77	20.61	0.79	0.028	4.81
TG (16:0/16:0/18:1) + NH4	TG (50:1) + NH4	C53 H104 O6 N1	850.79	21.64	0.83	0.014	4.80
TG (18:0/16:0/16:0) + NH4	TG (50:0) + NH4	C53 H106 O6 N1	852.80	22.48	0.78	0.011	4.74
TG (18:0/16:0/18:1) + NH4	TG (52:1) + NH4	C55 H108 O6 N1	878.82	22.48	0.83	0.046	4.71

### Identification and cluster analysis of potential lipid molecule markers

**Figure 6:**
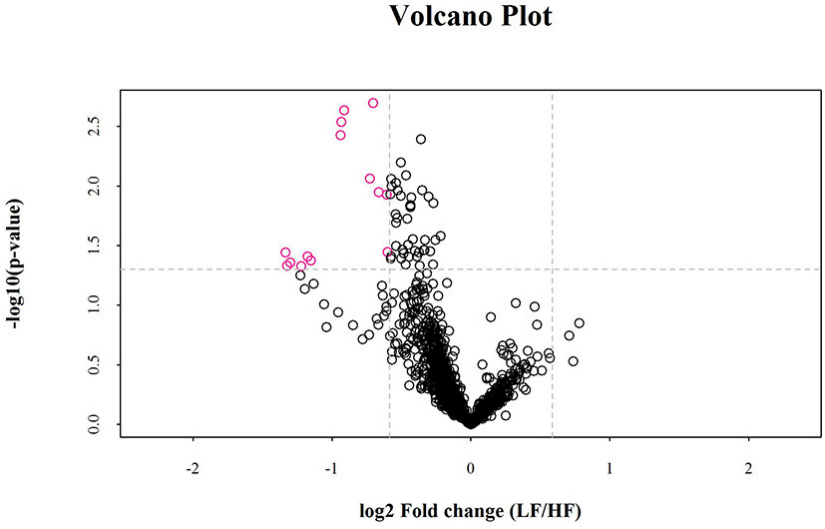
Volcano plots for the distribution of significantly differences in lipid molecules. The rosy dots are significantly differential lipid molecules screened for univariate statistical analysis.

**Figure 7:**
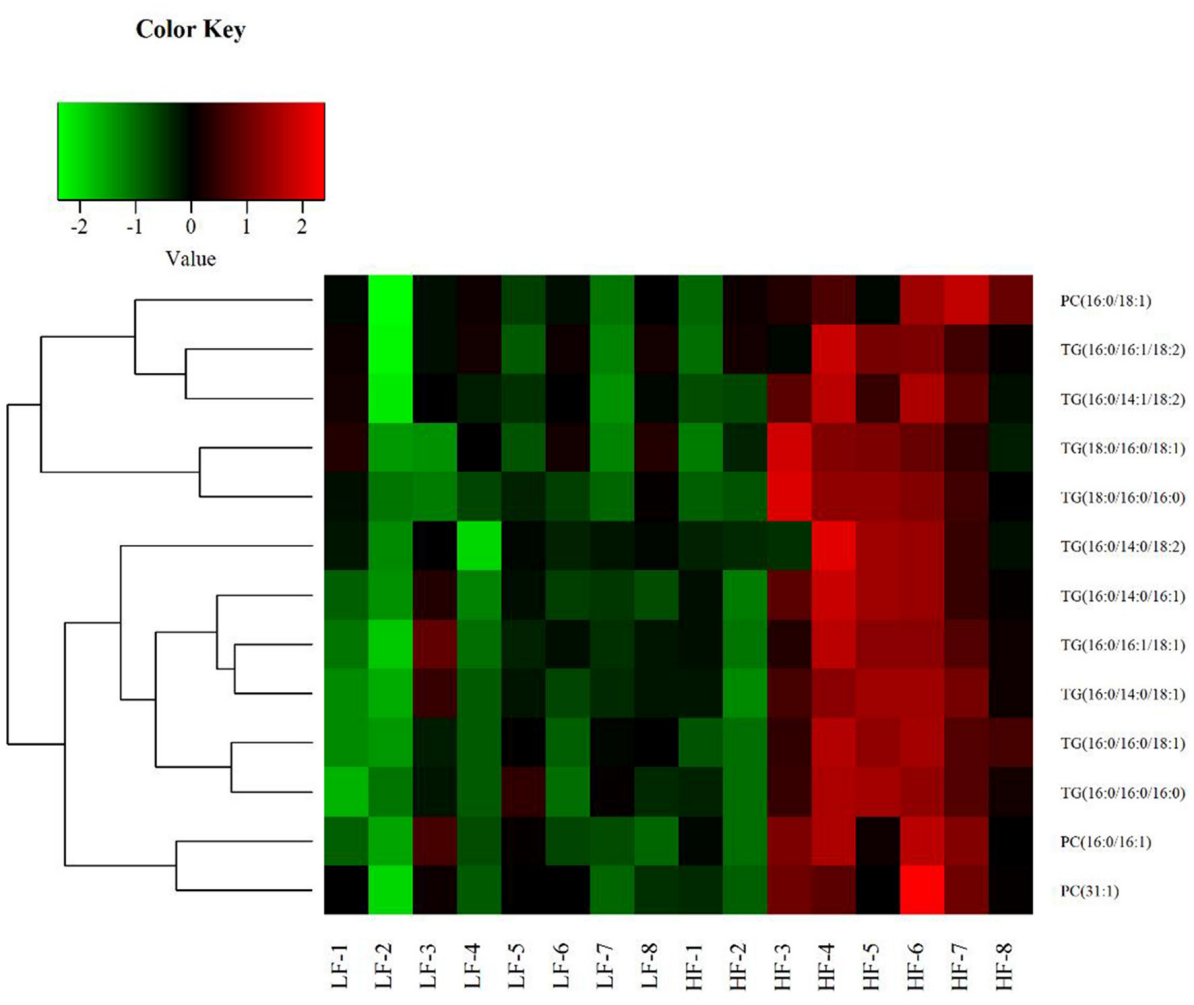
Hierarchical Clustering results of significant differential lipid molecules. The greenpart represents down regulation of expression. The red part represents upregulation.

Univariate statistical analyses, including fold change analysis and t-test, were used to visualize the significance of lipid changes between the HF and LF sperm after cryopreservation, which can screen for potential lipid molecular markers. A volcano plot was drawn using an FC > 1.5 or FC < 0.67 and *P* < 0.05 as the screening criteria (Figure 6), to assess the abundance of differential lipids and illustrate the connection between samples and variations in lipid expression patterns. To evaluate the rationality of differential lipids and visualize the relationship between samples and the differences in lipid expression patterns, the samples were hierarchically clustered based on the expression of significantly differential lipids molecular (VIP>1, *P* < 0.05). It was observed that the lipid expression profiles could be clearly divided into two categories (Figure 7). Therefore, it was indicated that sperm from HF and LF groups in goats showed significantly differential lipid expression patterns after cryopreservation.

## Discussion

Semen cryopreservation causes the sperm to undergo considerable biophysical and biochemical changes, including alterations to membranes and their constituent parts, resulting in decreased post-thaw sperm motility and fertilization ability (36, 37). However, semen from different individuals had the various capacity to protect against freezing damage (38–41). In this study, the extent of cryopreservation-induced changes in motility to characterize variation in sperm cryotolerance across different individuals were measured. It was found that the quality of post-thawed sperm from different individuals varies greatly in motion parameter and structural integrity after a unified cryopreservation procedure. For 20 goats studied, eight goats were classified as high freezers group, and the sperm quality after thawed were better. The sperm from another eight goats showed very low quality after cryopreservation. However, the mechanism of the sperm cryotolerance among different individuals was not explained from now on. Such differences could be due to genetic and non-genetic factors. In origin, and making genetic selection of males as an important factor for successful sperm freezing (42). In previous studies, it attempted to elucidate differences in sperm cryotolerance through investigating marker prediction, management system and cryopreservation technology processes (43, 44). Thus, it is the first time to characterize the lipidomic profiles of post-thawed sperm with high and low freezing resistance, and investigate the relationship between the lipids of the sperm and the post-thawing sperm characteristics of goats.

As the main components of sperm plasma membranes, the lipids play an important role in regulating plasma membrane function (17, 45). The lipid composition of the sperm plasma membrane was susceptible to alteration during cryopreservation, affecting sperm motility and fertilization potential (45). Lipidomics is a rapid and sensitive tool for systematically resolving changes in lipid composition and expression in sperm (46). It was found that the lipids of sperm after freeze-thawed contained a large amount of the lipid subclass, such as phosphatidylcholine (PC), phosphatidethanolamine (PE), triglycerides (TG), ceramides (CER), sphingolipids (SM). Compared with LF group, the total lipids in the thawed sperm from HF was higher. Sperm with high and low cryotolerance had different heritability, transcripts, proteins, and metabolites associated with sperm function (47–50). The lipidomic analysis of post-thawed sperm in this study revealed that the lipid metabolism patterns with different cryotolerance were various, and the lipid molecules of PC and TG were significantly different between the two groups.

The higher relative abundances of phosphatidylcholines was observed in high Freezer group, represented by ions of m/z 718.54, 732.55, and 760.59. According to previous research, a positive correlation between phosphatidylcholines abundance and the sperm motility in thawed sperm had been found (12, 51). As the biomarkers related to sperm freezability, PC may affect the motility, membrane integrity and lipid peroxidation (13, 52). As the primary component of the plasma membrane, phosphatidylcholines are crucial for controlling the function of the membrane, particularly membrane fusion, fluidity and protein binding (53). During the cryopreservation of goat semen, the addition of a substance rich in phosphatidylcholines prevents sperm from suffering from decreased motility caused by cold shock as well as structural damage (54–56). When adding sperm freezing solution, isolated soybean phosphatidylcholine displays cryoprotective properties comparable to those of egg yolk. Compared to phosphatidylcholine or egg yolk alone, egg yolk and soy-phosphatidylcholine are added in combination have been found to support better post-thaw outcomes (57). The PC molecules were amphipathic. It could be incorporated into sperm membrane for preventing mechanical damage to membrane structures during cryopreservation processes, also be taken up and utilized during lipid metabolism (58). However, the PC supplement interacts with sperm membrane influencing the lipid profile of the post-thawed sperm is still unclear, it needed to be further studied.

In our study, the thawed HF sperm had a higher triglyceride content. However, triglycerides do not act as lipid components in plasma membranes, and the adverse effects of high levels of triglycerides on sperm have been described. The study showed that rats with diabetes exhibit decreased sperm motility and antioxidant enzyme activity and increased triglyceride levels in sperm (59). In infertility mice subjected to Slc22a14 (riboflavin transporter) knockout, triglycerides accumulate in sperm, and significant fatty acid β-oxidation deficiencies are observed. Additionally, levels of in acylcarnitines and metabolites from the TCA cycle were significantly reduced (60). In this study, the thawed sperm in HF group showed better quality before freezing. Therefore, the increase of the relative abundance of triglyceride lipid molecules after thawing may be due to that the sperm uptake lipids in the freezing diluent during cryopreservation. The previous studies reported that exogenous lipids can bind to the surface of the sperm plasma membrane, and provide a physical barrier to cryogenic damage by changing the lipid composition of the membrane (61, 62). Exogenous lipids are exchanged between the medium and the membrane, replenishing the lipids lost and stabilizing cold shock (24, 63). The thawed boar sperm with better quality may have a stronger affinity for yolk lipoprotein. The lipid content of the post-thawed sperm was increased, and was rich in triglycerides and phosphatidylcholine (64). The lipid synthesis capacity of mammalian sperm had been described in previous studies (65), and describing the synthesis of triglycerides have not been elaborated.

## Conclusions

In summary, the differences in the cryotolerance of sperm among individual goats were found. And it was observed that the total lipid content in the high freezer sperm after thawed is higher than that in the LF sperm, especially phosphatidylcholine and triglycerides subclass molecules. It may be that the high freezer sperm were able to take up more lipids in freezing diluent and may have the active lipid anabolic processes. But more research is needed to further understand the mechanism of lipid uptake in goat sperm and its relationship with the quality of goat sperm after thawed to improve preservation methods.

## Data availability statement

The datasets presented in this study can be found in online repositories. The names of the repository/repositories and accession number(s) can be found in the article/supplementary material.

## Ethics statement

The animal study was reviewed and approved by the Animal Ethics Committee of the Inner Mongolia Academy of Agriculture and Animal Husbandry Sciences. Written informed consent was obtained from the owners for the participation of their animals in this study.

## Author contributions

BX, RW, and ZhiW conceptualized the study. BX, RW, ZhiW, JL, and JZ conceptualized, designed and carried out the investigations. BX, HL, ZheW, and WZ performed the experiments. BX, YZ, RS, YL, and ZL analyzed the data. BX wrote the paper. JZ and JL critically revised the manuscript. All authors contributed to the article and approved the submitted version.

## Funding

This work was supported by the Major Projects of the Inner Mongolia Autonomous Region of China (No. 2020ZD0003), Inner Mongolia Autonomous Region Science and Technology Research Project (No. 2021GG0086), and China Agriculture Research System of MOF and MARA (No. CARS-39-06).

## Conflict of interest

Author JL was employed by the company Inner Mongolia Jinlai Livestock Technology Co., Ltd. The remaining authors declare that the research was conducted in the absence of any commercial or financial relationships that could be construed as a potential conflict of interest.

## Publisher's note

All claims expressed in this article are solely those of the authors and do not necessarily represent those of their affiliated organizations, or those of the publisher, the editors and the reviewers. Any product that may be evaluated in this article, or claim that may be made by its manufacturer, is not guaranteed or endorsed by the publisher.
